# 1-(Diphenyl­phosphinothio­yl)-2-[(4-methyl­phen­yl)meth­oxy­meth­yl]ferrocene

**DOI:** 10.1107/S1600536810040791

**Published:** 2010-10-20

**Authors:** Jean-Claude Daran, Catherine Audin, Eric Deydier, Eric Manoury, Rinaldo Poli

**Affiliations:** a Laboratoire de Chimie de Coordination, UPR-8241 CNRS, 205 route de Narbonne, 31077 Toulouse Cedex, France; b Université de Toulouse, UPS, Institut Universitaire de Technologie Paul Sabatier, Département de Chimie, Av. Georges Pompidou, F-81104 Castres Cedex, France

## Abstract

Following our continuing inter­est in developing new chiral phosphine-containing ferrocenyl ligands, we synthesized the title compound, [Fe(C_5_H_5_)(C_26_H_24_OPS)], in which there are two nearly identical mol­ecules in the asymmetric unit. The conformation of the cyclo­penta­dienyl (Cp) rings in each ferrocenyl group are inter­mediate between eclipsed and staggered, with twist angles of 16.6 (2) and 8.9 (2)°. The protecting S atom is located *endo* with respect to the substituted Cp ring. In the crystal, mol­ecules are connected through inter­molecular C—H⋯π inter­actions.

## Related literature

For background to homogenous asymmetric catalysis by transition metals, see: Collins *et al.* (1992 ▶); Jacobsen *et al.* (1999 ▶); Hawkins & Watson (2004 ▶); Blaser *et al.* (2007 ▶); Börner (2008 ▶). For the design and use of new chiral ligands, see: Atkinson *et al.* (2004 ▶); Audin *et al.* (2009 ▶); Breit & Breuniger (2004 ▶, 2005 ▶); Diab *et al.* (2008 ▶); Labande *et al.* (2007 ▶); Le Roux *et al.* (2007 ▶); Lopez Cortes *et al.* (2006 ▶); Manoury *et al.* (2000 ▶); Mateus *et al.* (2006 ▶); Mourgues *et al.* (2003 ▶); Routaboul *et al.* (2005 ▶, 2007 ▶); Teo *et al.* (2006 ▶); Yoshida & Itami (2002 ▶); Yu *et al.* (2007 ▶).
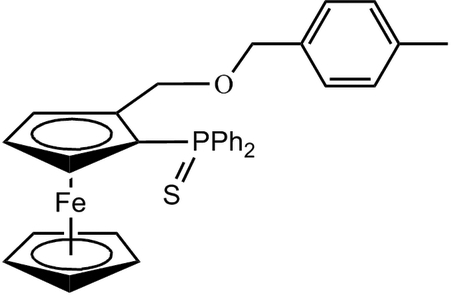


## Experimental

### 

#### Crystal data

[Fe(C_5_H_5_)(C_26_H_24_OPS)]
*M* *_r_* = 536.43
Triclinic, 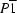

*a* = 9.0614 (4) Å
*b* = 14.9924 (8) Å
*c* = 19.118 (1) Å
α = 78.192 (3)°
β = 88.526 (3)°
γ = 86.917 (3)°
*V* = 2538.3 (2) Å^3^
*Z* = 4
Mo *K*α radiation
μ = 0.76 mm^−1^
*T* = 180 K
0.38 × 0.13 × 0.04 mm

#### Data collection

Bruker APEXII CCD area-detector diffractometer
Absorption correction: multi-scan (*SADABS*; Bruker, 2007 ▶) *T*_min_ = 0.708, *T*_max_ = 1.0
38324 measured reflections
8800 independent reflections
6532 reflections with *I* > 2σ(*I*)
*R*_int_ = 0.050

#### Refinement

*R*[*F* ^2^ > 2σ(*F* ^2^)] = 0.041
*wR*(*F* ^2^) = 0.108
*S* = 1.03
8800 reflections
631 parameters
H-atom parameters constrained
Δρ_max_ = 0.47 e Å^−3^
Δρ_min_ = −0.35 e Å^−3^

### 

Data collection: *APEX2* (Bruker, 2007 ▶); cell refinement: *SAINT* (Bruker, 2007 ▶); data reduction: *SAINT*; program(s) used to solve structure: *SIR97* (Altomare *et al.*, 1999 ▶); program(s) used to refine structure: *SHELXL97* (Sheldrick, 2008 ▶); molecular graphics: *ORTEPIII* (Burnett & Johnson, 1996 ▶), *ORTEP-3 for Windows* (Farrugia, 1997 ▶) and *PLATON* (Spek, 2009 ▶); software used to prepare material for publication: *WinGX* (Farrugia, 1999 ▶).

## Supplementary Material

Crystal structure: contains datablocks I, global. DOI: 10.1107/S1600536810040791/jh2205sup1.cif

Structure factors: contains datablocks I. DOI: 10.1107/S1600536810040791/jh2205Isup2.hkl

Additional supplementary materials:  crystallographic information; 3D view; checkCIF report

## Figures and Tables

**Table 1:** Hydrogen-bond geometry (Å, °) *Cg*1 and *Cg*2 are the centroids of the C111–C116 and C211–C216 rings, respectively.

*D*—H⋯*A*	*D*—H	H⋯*A*	*D*⋯*A*	*D*—H⋯*A*
C312—H32⋯*Cg*1	0.95	2.82	3.703 (3)	156
C123—H123⋯*Cg*2^i^	0.95	2.91	3.741 (4)	147

Symmetry code: (i) 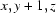.

**Fig. 1.**
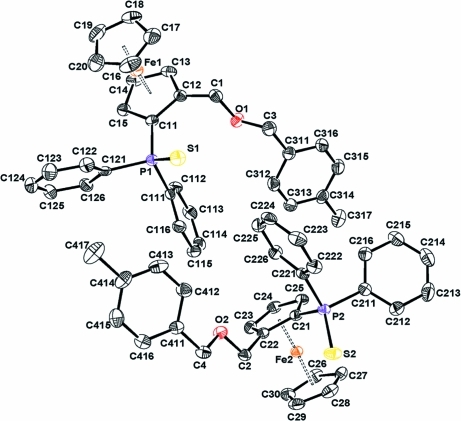
Molecular view of compound (I) showing 30% probability displacement ellipsoids and the atom numbering scheme. H atoms have been omitted for clarity.

**Fig. 2.**
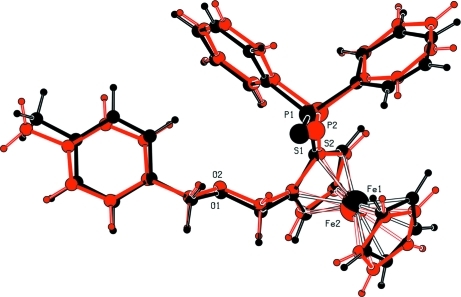
Molecular fitting of the two independent molecules building up the asymmetric unit.

**Fig. 3.**
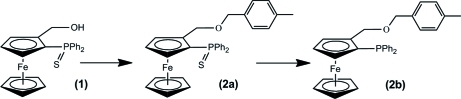
The formation of the title compound.

## supplementary crystallographic information

### Comment

Homogenous asymmetric catalysis by transition metals has received considerable attention over the last few decades, and numerous chiral ligands and complexes allowing high efficiency reactions have been reported (Jacobsen *et al.*, 1999; Börner, 2008). In this field, chiral phosphines have played a significant role. The possibility to easily modify their electronic and steric properties by a judicious choice of their substituents has proven extremely useful to successfully optimized catalytic reactions. However, only few examples have been transferred to industrial processes (Collins *et al.*, 1992;Hawkins & Watson, 2004; Blaser *et al.*, 2007) in many cases because of the expenses associated to ligand and catalyst loss.

The efficient separation of expensive catalysts and ligands to enable reuse in subsequent cycles is a main challenge that meets both industrial economical needs and new stricter environmental regulations. We have long been interested in the design and the synthesis of new chiral catalysts for exploring new asymmetric catalytic reactions or for improving existing ones (Manoury *et al.*, 2000; Mourgues *et al.*, 2003; Routaboul *et al.*, 2005; Lopez Cortes *et al.*, 2006; Mateus *et al.*, 2006; Routaboul *et al.*, 2007; Le Roux *et al.*, 2007; Labande *et al.*, 2007; Diab *et al.*, 2008). Among the numerous phosphine ligands reported to date, ferrocenyl phosphines functionalized by an oxygen atom (PO ferrocenyl phosphines) constitute a distinct class of hemilabile ligands attracting increasing interest (Breit & Breuniger, 2004; Atkinson *et al.*, 2004; Breit & Breuniger, 2005; Teo *et al.*, 2006; Yu *et al.*, 2007; Mateus *et al.*, 2006). We have recently developed promising PO ferrocenyl ligands (Mateus *et al.*, 2006; Audin *et al.*, 2009). In addition, we recently took interest in improving catalyst recycling using ionic liquid, water or PEG as catalyst "liquid carriers" and in investigating the influence of these media on both the catalytic reaction and the recycling efficiency. To reach this goal, we have prepared a new family of PO ferrocenyl phosphine-ethers, bearing charged (imidazolium) or neutral (monomethylether PEG 750, tetraethylbisphosphonate) polar tags (Audin *et al.*, 2009) to increase their solubility in non conventional media. The simplest member of this new family is compound 2 b (Scheme 1) which efficiency in the Suzuki-Miyaura reaction has been demonstrated (Yoshida & Itami, 2002).

The title molecule 2a is built up from a ferrocenyl moiety in which one Cp ring is substituted by a sulfur protected phosphine and a ((4-methylphenyl)-methoxy)methyl group resulting in a planar chirality. As the space group is centrosymmetric the two enantiomers *R*/S are present in the crystal (Fig. 1). There are two molecules with the same configuration within the asymmetric unit. As shown by molecular fitting (Spek, 2009), the two molecules have very closely related geometry (Fig. 2).

The ether chains are roughly planar with the largest deviation being 0.034 (2)Å at C4 and 0.100 (2)Å at O1. These planes makes dihedral angle of 83.21 (13) ° or 88.03 (13)° for molecule 1 and 2 respectively. The benzyl groups are twisted with respect to these plane by dihedral angle of 49.21 (17) ° and 33.04 (23)° for molecule 1 and 2 respectively.

The Cp rings within the ferrocene moiety have intermediate conformation between eclipsed and staggered with a twist angle of 16.6 (2)° and 8.9 (2)° respectively. These Cp rings are slightly bent wit respect to each other making dihedral angles of 2.16 (24)° and 4.06 (21)° respectively. The S atom is *endo* with respect to the Cp ring by 0.884 (7)Å and 0.992 (6)Å respectively.

There are weak intra and intermolecular C—H···π interactions involving H atoms of phenyl rings and related phenyl rings either to the same molecule or to a symmetry related one (Table 1, *Cg*1 is the centroid of the C111—C116 ring whereas *Cg*2 is the centroid of the C211—C216 ring).

### Experimental

In a Schlenk tube, 0.75 g of the racemic 2-thiodiphenylphosphino(hydroxymethyl)ferrocene (1.74 mmol) was dissolved in 8 ml of dry dichloromethane. A 54% solution of tetrafluoroboric acid in ether (0.73 ml, 5.30 mmol) was then added. After 1 min stirring, a solution of 2.5 g of 4-methylbenzylalcohol (20.5 mmol) in 8 mL of dry dichloromethane was added. After 1 min of stirring, the crude material was filtered on silica gel with ether as eluent. After evaporation of the solvent, 0.4 g of 2a was obtained as a yellow solid (yield = 43%). ^1^H NMR (200.1 MHz, CDCl_3_), d (p.p.m.): 7.90–7.65 (4*H*, m: PPh_2_); 7.52–7.34 (6*H*, m: PPh_2_); 7.05 (2*H*, d, J = 7.9 Hz: Ph); 6.95 (2*H*, d, J = 7.9 Hz: Ph); 4.89 (1*H*, d, J = 10.9 Hz: CpCH_2_); 4.66 (1*H*, m: subst Cp); 4.45 (1*H*, d, J = 10.9 Hz: CpCH_2_); 4.35 (1*H*, m: subst Cp); 4.33 (5*H*, s: Cp); 4.29 (2*H*, d: 2.9 Hz: PhCH_2_O); 3.85 (1*H*, m: subst Cp); 2.33 (3*H*, m: CH_3_). ^13^C NMR (50.3 MHz, CDCl_3_), d (p.p.m.): 136.8 (s: quat Ph); 135.4 (s: quat Ph); 134.8 (d, J_PC_ = 87.1 Hz: quat PPh_2_); 133.6 (d, J_PC_ = 86.1 Hz: quat PPh_2_); 132.2 (d, J_PC_ = 10.7 Hz: PPh_2_); 132.1 (d, J_PC_ = 10.7 Hz: PPh_2_); 131.2 (d, J_PC_ = 2.2 Hz: PPh_2_); 131.1 (d, J_PC_ = 2.2 Hz: PPh_2_); 128.8 (s: Ph); 128.1 (d, J_PC_ = 12.3 Hz: PPh_2_); 128.0 (d, J_PC_ = 12.3 Hz: PPh_2_); 127.7 (s: Ph); 88.3 (d, J_PC_ = 12.1 Hz: quat Cp); 75.2 (d, J_PC_ = 12.5 Hz: subst Cp); 75.6 (d, J_PC_ = 94.7 Hz: quat Cp); 74.5 (d, J_PC_ = 9.4 Hz: subst Cp); 72.4 (s, PhCH_2_O); 70.7 (s: Cp); 69.4 (d, J_PC_ = 10.4 Hz: subst Cp); 66.7 (s: CpCH_2_O); 21.3 (s: CH_3_). ^31^P NMR (81.0 MHz, CDCl_3_), d (p.p.m.): 43.1. HR MS (DCI CH_4_), C_31_H_29_OPSFe, calcd. mass [*M*]: 536.1026; exp. mass [*M*]: 536.1039.

### Refinement

All H atoms attached to carbon were fixed geometrically and treated as riding with C—H = 0.98 Å (methyl) or 0.99 Å (methylene) and 0.97Å (methyl) with *U*_iso_(H) = 1.2*U*_eq_(C) or *U*_iso_(H) = 1.5*U*_eq_(methyl).

### Crystal data

**Table:**

[Fe(C_5_H_5_)(C_26_H_24_OPS)]	*Z* = 4
*M**_r_* = 536.43	*F*(000) = 1120
Triclinic, *P*1	*D*_x_ = 1.404 Mg m^−^^3^
Hall symbol: -P 1	Mo *K*α radiation, λ = 0.71073 Å
*a* = 9.0614 (4) Å	Cell parameters from 9957 reflections
*b* = 14.9924 (8) Å	θ = 1.6–26.0°
*c* = 19.118 (1) Å	µ = 0.76 mm^−^^1^
α = 78.192 (3)°	*T* = 180 K
β = 88.526 (3)°	Flattened, yellow
γ = 86.917 (3)°	0.38 × 0.13 × 0.04 mm
*V* = 2538.3 (2) Å^3^	

### Data collection

**Table:**

Bruker APEXII CCD area-detector diffractometer	8800 independent reflections
Radiation source: sealed tube	6532 reflections with *I* > 2σ(*I*)
graphite	*R*_int_ = 0.050
φ and ω scans	θ_max_ = 25.0°, θ_min_ = 1.1°
Absorption correction: multi-scan (*SADABS*; Bruker, 2007)	*h* = −10→10
*T*_min_ = 0.708, *T*_max_ = 1.0	*k* = −17→17
38324 measured reflections	*l* = −22→22

### Refinement

**Table:**

Refinement on *F*^2^	Primary atom site location: structure-invariant direct methods
Least-squares matrix: full	Secondary atom site location: difference Fourier map
*R*[*F*^2^ > 2σ(*F*^2^)] = 0.041	Hydrogen site location: inferred from neighbouring sites
*wR*(*F*^2^) = 0.108	H-atom parameters constrained
*S* = 1.03	*w* = 1/[σ^2^(*F*_o_^2^) + (0.0519*P*)^2^ + 1.7686*P*] where *P* = (*F*_o_^2^ + 2*F*_c_^2^)/3
8800 reflections	(Δ/σ)_max_ = 0.001
631 parameters	Δρ_max_ = 0.47 e Å^−^^3^
0 restraints	Δρ_min_ = −0.35 e Å^−^^3^

### Special details

**Table:**

Geometry. All e.s.d.'s (except the e.s.d. in the dihedral angle between two l.s. planes) are estimated using the full covariance matrix. The cell e.s.d.'s are taken into account individually in the estimation of e.s.d.'s in distances, angles and torsion angles; correlations between e.s.d.'s in cell parameters are only used when they are defined by crystal symmetry. An approximate (isotropic) treatment of cell e.s.d.'s is used for estimating e.s.d.'s involving l.s. planes.
Refinement. Refinement of *F*^2^ against ALL reflections. The weighted *R*-factor *wR* and goodness of fit *S* are based on *F*^2^, conventional *R*-factors *R* are based on *F*, with *F* set to zero for negative *F*^2^. The threshold expression of *F*^2^ > σ(*F*^2^) is used only for calculating *R*-factors(gt) *etc*. and is not relevant to the choice of reflections for refinement. *R*-factors based on *F*^2^ are statistically about twice as large as those based on *F*, and *R*- factors based on ALL data will be even larger.

### Fractional atomic coordinates and isotropic or equivalent isotropic displacement parameters (Å^2^)

**Table:**

	*x*	*y*	*z*	*U*_iso_*/*U*_eq_	
Fe1	0.86195 (5)	0.76305 (3)	0.43370 (2)	0.02412 (13)	
S1	0.46967 (9)	0.68675 (6)	0.34969 (4)	0.0296 (2)	
P1	0.66077 (9)	0.70617 (5)	0.29960 (4)	0.01973 (18)	
O1	0.7652 (2)	0.48562 (14)	0.42809 (11)	0.0265 (5)	
C11	0.8218 (3)	0.6972 (2)	0.35406 (15)	0.0211 (7)	
C12	0.8532 (3)	0.6330 (2)	0.42012 (15)	0.0221 (7)	
C13	0.9978 (4)	0.6477 (2)	0.44049 (16)	0.0277 (7)	
H13	1.0474	0.6143	0.4816	0.033*	
C14	1.0566 (4)	0.7201 (2)	0.38963 (16)	0.0304 (8)	
H14	1.1513	0.7439	0.3911	0.036*	
C15	0.9489 (3)	0.7511 (2)	0.33610 (16)	0.0249 (7)	
H15	0.9592	0.7990	0.2955	0.030*	
C16	0.6693 (4)	0.8230 (3)	0.4632 (2)	0.0437 (10)	
H16	0.5733	0.8142	0.4477	0.052*	
C17	0.7395 (4)	0.7716 (2)	0.52380 (19)	0.0382 (9)	
H17	0.6997	0.7220	0.5565	0.046*	
C18	0.8798 (4)	0.8065 (3)	0.52788 (18)	0.0395 (9)	
H18	0.9513	0.7842	0.5635	0.047*	
C19	0.8949 (5)	0.8799 (3)	0.4698 (2)	0.0475 (10)	
H19	0.9781	0.9166	0.4597	0.057*	
C20	0.7653 (5)	0.8900 (3)	0.4292 (2)	0.0500 (11)	
H20	0.7461	0.9339	0.3866	0.060*	
C1	0.7525 (4)	0.5638 (2)	0.45980 (15)	0.0254 (7)	
H36A	0.7803	0.5469	0.5107	0.030*	
H36B	0.6492	0.5893	0.4571	0.030*	
C3	0.6495 (4)	0.4250 (2)	0.45102 (17)	0.0313 (8)	
H37A	0.5524	0.4563	0.4375	0.038*	
H37B	0.6508	0.4043	0.5036	0.038*	
C111	0.6993 (3)	0.62643 (19)	0.24091 (15)	0.0216 (7)	
C112	0.8372 (4)	0.5835 (2)	0.23597 (16)	0.0262 (7)	
H112	0.9173	0.5975	0.2622	0.031*	
C113	0.8583 (4)	0.5199 (2)	0.19264 (16)	0.0314 (8)	
H113	0.9531	0.4907	0.1892	0.038*	
C114	0.7422 (4)	0.4988 (2)	0.15454 (16)	0.0303 (8)	
H114	0.7564	0.4539	0.1261	0.036*	
C115	0.6057 (4)	0.5429 (2)	0.15788 (16)	0.0305 (8)	
H115	0.5265	0.5297	0.1306	0.037*	
C116	0.5838 (4)	0.6062 (2)	0.20070 (15)	0.0263 (7)	
H116	0.4893	0.6363	0.2029	0.032*	
C121	0.6664 (3)	0.8185 (2)	0.24244 (15)	0.0210 (7)	
C122	0.5694 (4)	0.8880 (2)	0.25657 (17)	0.0298 (8)	
H122	0.4989	0.8754	0.2945	0.036*	
C123	0.5750 (4)	0.9750 (2)	0.21573 (18)	0.0365 (9)	
H123	0.5088	1.0222	0.2258	0.044*	
C124	0.6769 (4)	0.9935 (2)	0.16011 (18)	0.0377 (9)	
H124	0.6820	1.0537	0.1327	0.045*	
C125	0.7710 (4)	0.9245 (2)	0.14449 (17)	0.0320 (8)	
H125	0.8395	0.9370	0.1057	0.038*	
C126	0.7657 (4)	0.8369 (2)	0.18535 (16)	0.0276 (7)	
H126	0.8301	0.7894	0.1743	0.033*	
C311	0.6746 (3)	0.3453 (2)	0.41541 (16)	0.0265 (7)	
C312	0.6859 (4)	0.3588 (2)	0.34178 (17)	0.0294 (8)	
H32	0.6723	0.4188	0.3140	0.035*	
C313	0.7166 (4)	0.2868 (2)	0.30777 (17)	0.0290 (8)	
H33	0.7233	0.2979	0.2571	0.035*	
C314	0.7379 (4)	0.1983 (2)	0.34682 (17)	0.0294 (8)	
C315	0.7238 (4)	0.1845 (2)	0.42090 (19)	0.0387 (9)	
H35	0.7354	0.1244	0.4487	0.046*	
C316	0.6934 (4)	0.2567 (2)	0.45458 (18)	0.0356 (9)	
H38	0.6853	0.2456	0.5052	0.043*	
C317	0.7759 (4)	0.1206 (2)	0.3098 (2)	0.0430 (9)	
H30A	0.8552	0.0812	0.3355	0.064*	
H30B	0.8089	0.1448	0.2607	0.064*	
H30C	0.6884	0.0851	0.3091	0.064*	
Fe2	0.42478 (5)	0.23170 (3)	0.05815 (2)	0.02081 (12)	
S2	0.01485 (9)	0.26601 (6)	0.15842 (4)	0.0312 (2)	
P2	0.20642 (9)	0.26407 (5)	0.20415 (4)	0.02038 (18)	
O2	0.2595 (2)	0.50360 (14)	0.07561 (11)	0.0254 (5)	
C21	0.3631 (3)	0.2845 (2)	0.14494 (15)	0.0203 (7)	
C22	0.3757 (3)	0.35567 (19)	0.08177 (15)	0.0215 (7)	
C23	0.5249 (3)	0.3510 (2)	0.05618 (16)	0.0255 (7)	
H23	0.5650	0.3900	0.0155	0.031*	
C24	0.6032 (3)	0.2784 (2)	0.10168 (16)	0.0264 (7)	
H24	0.7043	0.2603	0.0964	0.032*	
C25	0.5056 (3)	0.2372 (2)	0.15631 (15)	0.0217 (7)	
H25	0.5299	0.1870	0.1940	0.026*	
C26	0.5004 (4)	0.1334 (2)	0.00378 (17)	0.0313 (8)	
H26	0.5984	0.1074	0.0043	0.038*	
C27	0.3842 (4)	0.1002 (2)	0.05133 (17)	0.0312 (8)	
H27	0.3903	0.0482	0.0892	0.037*	
C28	0.2568 (4)	0.1588 (2)	0.03218 (18)	0.0347 (8)	
H28	0.1626	0.1529	0.0551	0.042*	
C29	0.2949 (4)	0.2274 (2)	−0.02704 (17)	0.0337 (8)	
H29	0.2307	0.2756	−0.0509	0.040*	
C30	0.4457 (4)	0.2116 (2)	−0.04452 (16)	0.0317 (8)	
H30	0.5004	0.2474	−0.0821	0.038*	
C2	0.2585 (4)	0.4224 (2)	0.04715 (15)	0.0252 (7)	
H41A	0.2765	0.4374	−0.0051	0.030*	
H41B	0.1607	0.3954	0.0561	0.030*	
C4	0.1403 (4)	0.5657 (2)	0.04868 (16)	0.0259 (7)	
H42A	0.0452	0.5370	0.0626	0.031*	
H42B	0.1467	0.5814	−0.0041	0.031*	
C211	0.2497 (3)	0.1565 (2)	0.26664 (15)	0.0217 (7)	
C212	0.1717 (4)	0.0799 (2)	0.26451 (17)	0.0326 (8)	
H212	0.1015	0.0817	0.2282	0.039*	
C213	0.1970 (4)	0.0005 (2)	0.3159 (2)	0.0425 (9)	
H213	0.1425	−0.0516	0.3150	0.051*	
C214	0.3006 (4)	−0.0030 (2)	0.36819 (18)	0.0397 (9)	
H214	0.3182	−0.0577	0.4027	0.048*	
C215	0.3783 (4)	0.0726 (2)	0.37037 (17)	0.0348 (8)	
H215	0.4496	0.0700	0.4064	0.042*	
C216	0.3531 (4)	0.1524 (2)	0.32022 (16)	0.0271 (7)	
H216	0.4064	0.2047	0.3223	0.033*	
C221	0.2101 (3)	0.3491 (2)	0.25909 (15)	0.0209 (7)	
C222	0.0866 (4)	0.3630 (2)	0.30094 (16)	0.0273 (7)	
H222	0.0038	0.3267	0.3013	0.033*	
C223	0.0824 (4)	0.4289 (2)	0.34197 (16)	0.0317 (8)	
H223	−0.0027	0.4380	0.3702	0.038*	
C224	0.2031 (4)	0.4816 (2)	0.34168 (16)	0.0289 (8)	
H224	0.1996	0.5283	0.3687	0.035*	
C225	0.3280 (4)	0.4669 (2)	0.30249 (16)	0.0303 (8)	
H225	0.4118	0.5019	0.3039	0.036*	
C226	0.3321 (4)	0.4010 (2)	0.26091 (16)	0.0266 (7)	
H226	0.4184	0.3913	0.2336	0.032*	
C411	0.1469 (3)	0.6503 (2)	0.07826 (16)	0.0231 (7)	
C412	0.1922 (4)	0.6465 (2)	0.14800 (17)	0.0300 (8)	
H44	0.2183	0.5892	0.1777	0.036*	
C413	0.1995 (4)	0.7254 (2)	0.17434 (18)	0.0339 (8)	
H45	0.2301	0.7212	0.2221	0.041*	
C414	0.1630 (4)	0.8110 (2)	0.13251 (19)	0.0333 (8)	
C415	0.1155 (4)	0.8136 (2)	0.06389 (18)	0.0341 (8)	
H200	0.0878	0.8709	0.0345	0.041*	
C416	0.1070 (3)	0.7353 (2)	0.03680 (17)	0.0279 (7)	
H100	0.0737	0.7397	−0.0105	0.033*	
C417	0.1770 (5)	0.8963 (3)	0.1613 (2)	0.0545 (11)	
H40A	0.1465	0.9497	0.1248	0.082*	
H40B	0.2800	0.9011	0.1740	0.082*	
H40C	0.1136	0.8938	0.2039	0.082*	

### Atomic displacement parameters (Å^2^)

**Table:**

	*U*^11^	*U*^22^	*U*^33^	*U*^12^	*U*^13^	*U*^23^
Fe1	0.0259 (3)	0.0252 (3)	0.0226 (2)	−0.0008 (2)	−0.00231 (19)	−0.00780 (19)
S1	0.0216 (5)	0.0388 (5)	0.0292 (4)	−0.0049 (4)	0.0050 (3)	−0.0089 (4)
P1	0.0184 (4)	0.0218 (4)	0.0193 (4)	−0.0014 (3)	−0.0002 (3)	−0.0049 (3)
O1	0.0250 (13)	0.0276 (12)	0.0287 (12)	−0.0059 (10)	0.0066 (9)	−0.0095 (9)
C11	0.0211 (18)	0.0234 (16)	0.0202 (15)	−0.0002 (13)	−0.0003 (13)	−0.0077 (12)
C12	0.0256 (18)	0.0223 (16)	0.0191 (15)	0.0036 (13)	−0.0003 (13)	−0.0068 (12)
C13	0.030 (2)	0.0319 (18)	0.0223 (16)	0.0082 (15)	−0.0069 (14)	−0.0111 (14)
C14	0.0200 (19)	0.045 (2)	0.0283 (17)	−0.0016 (15)	−0.0004 (14)	−0.0132 (15)
C15	0.0200 (18)	0.0311 (18)	0.0241 (16)	−0.0062 (14)	0.0007 (13)	−0.0056 (13)
C16	0.035 (2)	0.046 (2)	0.060 (3)	0.0126 (19)	−0.0070 (19)	−0.036 (2)
C17	0.046 (2)	0.037 (2)	0.036 (2)	−0.0020 (18)	0.0111 (17)	−0.0198 (16)
C18	0.043 (2)	0.051 (2)	0.0317 (19)	0.0027 (19)	−0.0051 (16)	−0.0243 (18)
C19	0.056 (3)	0.041 (2)	0.054 (2)	−0.021 (2)	0.011 (2)	−0.027 (2)
C20	0.077 (3)	0.027 (2)	0.047 (2)	0.007 (2)	−0.007 (2)	−0.0122 (17)
C1	0.0307 (19)	0.0253 (17)	0.0201 (15)	0.0034 (14)	0.0036 (14)	−0.0060 (13)
C3	0.029 (2)	0.038 (2)	0.0281 (17)	−0.0101 (16)	0.0063 (15)	−0.0069 (15)
C111	0.0297 (19)	0.0166 (15)	0.0168 (15)	−0.0017 (13)	0.0023 (13)	0.0001 (12)
C112	0.0257 (19)	0.0294 (18)	0.0242 (16)	0.0017 (14)	−0.0054 (14)	−0.0071 (13)
C113	0.036 (2)	0.0303 (19)	0.0264 (17)	0.0070 (15)	0.0019 (15)	−0.0040 (14)
C114	0.050 (2)	0.0236 (17)	0.0172 (15)	−0.0034 (16)	0.0055 (15)	−0.0038 (13)
C115	0.035 (2)	0.0347 (19)	0.0230 (17)	−0.0097 (16)	−0.0031 (14)	−0.0070 (14)
C116	0.0255 (19)	0.0305 (18)	0.0223 (16)	−0.0030 (14)	−0.0024 (14)	−0.0036 (13)
C121	0.0222 (18)	0.0240 (16)	0.0180 (15)	−0.0010 (13)	−0.0071 (13)	−0.0064 (12)
C122	0.028 (2)	0.0303 (19)	0.0313 (18)	0.0004 (15)	−0.0012 (15)	−0.0078 (15)
C123	0.042 (2)	0.0249 (19)	0.043 (2)	0.0091 (16)	−0.0039 (18)	−0.0092 (16)
C124	0.049 (2)	0.0237 (18)	0.038 (2)	−0.0025 (17)	−0.0110 (18)	−0.0006 (15)
C125	0.038 (2)	0.0305 (19)	0.0250 (17)	−0.0057 (16)	0.0009 (15)	0.0001 (14)
C126	0.033 (2)	0.0256 (18)	0.0233 (16)	0.0026 (15)	−0.0021 (14)	−0.0039 (13)
C311	0.0212 (18)	0.0327 (19)	0.0258 (17)	−0.0089 (14)	0.0021 (13)	−0.0047 (14)
C312	0.027 (2)	0.0284 (18)	0.0310 (18)	−0.0056 (15)	0.0014 (14)	−0.0001 (14)
C313	0.027 (2)	0.0344 (19)	0.0257 (17)	−0.0074 (15)	0.0000 (14)	−0.0040 (14)
C314	0.0223 (19)	0.0284 (18)	0.0375 (19)	−0.0073 (14)	−0.0033 (15)	−0.0053 (15)
C315	0.043 (2)	0.029 (2)	0.042 (2)	−0.0092 (17)	−0.0085 (17)	0.0020 (16)
C316	0.039 (2)	0.038 (2)	0.0276 (18)	−0.0175 (17)	−0.0044 (15)	0.0015 (15)
C317	0.044 (2)	0.032 (2)	0.054 (2)	−0.0075 (17)	0.0045 (19)	−0.0098 (17)
Fe2	0.0215 (3)	0.0226 (2)	0.0195 (2)	−0.00141 (19)	0.00035 (18)	−0.00701 (18)
S2	0.0193 (5)	0.0491 (5)	0.0269 (4)	−0.0025 (4)	−0.0034 (3)	−0.0111 (4)
P2	0.0193 (5)	0.0245 (4)	0.0181 (4)	−0.0012 (3)	0.0000 (3)	−0.0061 (3)
O2	0.0246 (13)	0.0229 (11)	0.0302 (12)	0.0032 (9)	−0.0063 (9)	−0.0090 (9)
C21	0.0213 (18)	0.0215 (16)	0.0196 (15)	0.0011 (13)	−0.0020 (13)	−0.0083 (12)
C22	0.0294 (19)	0.0196 (16)	0.0177 (15)	−0.0022 (13)	−0.0005 (13)	−0.0084 (12)
C23	0.028 (2)	0.0266 (17)	0.0236 (16)	−0.0067 (14)	0.0052 (14)	−0.0083 (13)
C24	0.0165 (18)	0.0342 (19)	0.0318 (18)	−0.0027 (14)	−0.0005 (14)	−0.0135 (15)
C25	0.0219 (18)	0.0243 (17)	0.0200 (15)	0.0014 (13)	−0.0018 (13)	−0.0078 (12)
C26	0.027 (2)	0.0341 (19)	0.0377 (19)	0.0014 (15)	0.0039 (15)	−0.0208 (16)
C27	0.041 (2)	0.0210 (17)	0.0335 (18)	−0.0057 (15)	−0.0043 (16)	−0.0087 (14)
C28	0.030 (2)	0.039 (2)	0.041 (2)	−0.0074 (16)	−0.0004 (16)	−0.0209 (17)
C29	0.041 (2)	0.0312 (19)	0.0321 (19)	0.0051 (16)	−0.0151 (16)	−0.0146 (15)
C30	0.046 (2)	0.0330 (19)	0.0197 (16)	−0.0035 (16)	0.0024 (15)	−0.0130 (14)
C2	0.034 (2)	0.0233 (17)	0.0189 (15)	−0.0001 (14)	−0.0033 (13)	−0.0054 (13)
C4	0.0236 (18)	0.0312 (18)	0.0235 (16)	0.0026 (14)	−0.0026 (13)	−0.0078 (14)
C211	0.0196 (17)	0.0253 (17)	0.0205 (15)	0.0008 (13)	0.0046 (13)	−0.0064 (13)
C212	0.037 (2)	0.0314 (19)	0.0319 (18)	−0.0060 (16)	−0.0019 (15)	−0.0103 (15)
C213	0.053 (3)	0.0263 (19)	0.049 (2)	−0.0114 (17)	0.0082 (19)	−0.0071 (17)
C214	0.054 (3)	0.030 (2)	0.0308 (19)	0.0054 (18)	0.0086 (18)	0.0011 (15)
C215	0.042 (2)	0.034 (2)	0.0264 (18)	0.0063 (17)	−0.0021 (15)	−0.0042 (15)
C216	0.029 (2)	0.0267 (18)	0.0267 (17)	−0.0016 (14)	0.0037 (14)	−0.0072 (14)
C221	0.0225 (18)	0.0216 (16)	0.0180 (15)	0.0031 (13)	−0.0034 (13)	−0.0031 (12)
C222	0.0210 (18)	0.0353 (19)	0.0254 (17)	−0.0007 (14)	0.0008 (14)	−0.0061 (14)
C223	0.030 (2)	0.042 (2)	0.0239 (17)	0.0103 (17)	0.0019 (14)	−0.0110 (15)
C224	0.042 (2)	0.0265 (18)	0.0183 (15)	0.0079 (16)	−0.0047 (15)	−0.0074 (13)
C225	0.038 (2)	0.0286 (18)	0.0255 (17)	−0.0055 (15)	0.0020 (15)	−0.0085 (14)
C226	0.0259 (19)	0.0282 (18)	0.0261 (17)	−0.0025 (14)	0.0040 (14)	−0.0072 (14)
C411	0.0138 (17)	0.0297 (18)	0.0265 (16)	−0.0002 (13)	−0.0001 (13)	−0.0079 (13)
C412	0.030 (2)	0.0301 (19)	0.0297 (18)	0.0055 (15)	−0.0062 (15)	−0.0060 (14)
C413	0.0233 (19)	0.049 (2)	0.0340 (19)	−0.0007 (16)	−0.0041 (15)	−0.0204 (17)
C414	0.0197 (19)	0.034 (2)	0.050 (2)	−0.0038 (15)	0.0027 (16)	−0.0179 (17)
C415	0.032 (2)	0.0278 (19)	0.040 (2)	0.0027 (15)	0.0062 (16)	−0.0032 (15)
C416	0.0229 (19)	0.0344 (19)	0.0257 (17)	0.0052 (15)	0.0004 (14)	−0.0065 (14)
C417	0.047 (3)	0.048 (3)	0.078 (3)	−0.011 (2)	0.003 (2)	−0.031 (2)

### Geometric parameters (Å, °)

**Table:**

Fe1—C12	2.024 (3)	Fe2—C22	2.025 (3)
Fe1—C11	2.027 (3)	Fe2—C21	2.034 (3)
Fe1—C20	2.038 (4)	Fe2—C23	2.042 (3)
Fe1—C16	2.040 (4)	Fe2—C29	2.048 (3)
Fe1—C15	2.044 (3)	Fe2—C30	2.049 (3)
Fe1—C17	2.047 (3)	Fe2—C28	2.049 (3)
Fe1—C18	2.049 (3)	Fe2—C26	2.050 (3)
Fe1—C19	2.049 (4)	Fe2—C25	2.051 (3)
Fe1—C13	2.052 (3)	Fe2—C27	2.055 (3)
Fe1—C14	2.063 (3)	Fe2—C24	2.056 (3)
S1—P1	1.9635 (11)	S2—P2	1.9598 (11)
P1—C11	1.797 (3)	P2—C21	1.792 (3)
P1—C121	1.812 (3)	P2—C221	1.813 (3)
P1—C111	1.815 (3)	P2—C211	1.830 (3)
O1—C1	1.424 (3)	O2—C4	1.422 (4)
O1—C3	1.425 (4)	O2—C2	1.432 (3)
C11—C15	1.436 (4)	C21—C25	1.439 (4)
C11—C12	1.447 (4)	C21—C22	1.445 (4)
C12—C13	1.417 (4)	C22—C23	1.430 (4)
C12—C1	1.493 (4)	C22—C2	1.489 (4)
C13—C14	1.419 (5)	C23—C24	1.416 (4)
C13—H13	0.9500	C23—H23	0.9500
C14—C15	1.423 (4)	C24—C25	1.415 (4)
C14—H14	0.9500	C24—H24	0.9500
C15—H15	0.9500	C25—H25	0.9500
C16—C17	1.399 (5)	C26—C30	1.411 (5)
C16—C20	1.410 (6)	C26—C27	1.416 (5)
C16—H16	0.9500	C26—H26	0.9500
C17—C18	1.410 (5)	C27—C28	1.420 (5)
C17—H17	0.9500	C27—H27	0.9500
C18—C19	1.404 (5)	C28—C29	1.414 (5)
C18—H18	0.9500	C28—H28	0.9500
C19—C20	1.409 (6)	C29—C30	1.418 (5)
C19—H19	0.9500	C29—H29	0.9500
C20—H20	0.9500	C30—H30	0.9500
C1—H36A	0.9900	C2—H41A	0.9900
C1—H36B	0.9900	C2—H41B	0.9900
C3—C311	1.496 (4)	C4—C411	1.495 (4)
C3—H37A	0.9900	C4—H42A	0.9900
C3—H37B	0.9900	C4—H42B	0.9900
C111—C112	1.384 (4)	C211—C212	1.388 (4)
C111—C116	1.396 (4)	C211—C216	1.396 (4)
C112—C113	1.388 (4)	C212—C213	1.393 (5)
C112—H112	0.9500	C212—H212	0.9500
C113—C114	1.381 (5)	C213—C214	1.379 (5)
C113—H113	0.9500	C213—H213	0.9500
C114—C115	1.377 (5)	C214—C215	1.375 (5)
C114—H114	0.9500	C214—H214	0.9500
C115—C116	1.378 (4)	C215—C216	1.384 (4)
C115—H115	0.9500	C215—H215	0.9500
C116—H116	0.9500	C216—H216	0.9500
C121—C126	1.389 (4)	C221—C226	1.391 (4)
C121—C122	1.392 (4)	C221—C222	1.391 (4)
C122—C123	1.379 (5)	C222—C223	1.379 (4)
C122—H122	0.9500	C222—H222	0.9500
C123—C124	1.385 (5)	C223—C224	1.382 (5)
C123—H123	0.9500	C223—H223	0.9500
C124—C125	1.381 (5)	C224—C225	1.375 (5)
C124—H124	0.9500	C224—H224	0.9500
C125—C126	1.386 (4)	C225—C226	1.387 (4)
C125—H125	0.9500	C225—H225	0.9500
C126—H126	0.9500	C226—H226	0.9500
C311—C312	1.383 (4)	C411—C416	1.392 (4)
C311—C316	1.389 (5)	C411—C412	1.394 (4)
C312—C313	1.383 (4)	C412—C413	1.383 (4)
C312—H32	0.9500	C412—H44	0.9500
C313—C314	1.388 (4)	C413—C414	1.394 (5)
C313—H33	0.9500	C413—H45	0.9500
C314—C315	1.392 (5)	C414—C415	1.384 (5)
C314—C317	1.503 (5)	C414—C417	1.505 (5)
C315—C316	1.381 (5)	C415—C416	1.383 (5)
C315—H35	0.9500	C415—H200	0.9500
C316—H38	0.9500	C416—H100	0.9500
C317—H30A	0.9800	C417—H40A	0.9800
C317—H30B	0.9800	C417—H40B	0.9800
C317—H30C	0.9800	C417—H40C	0.9800
			
C12—Fe1—C11	41.85 (11)	C22—Fe2—C21	41.71 (11)
C12—Fe1—C20	150.62 (16)	C22—Fe2—C23	41.16 (12)
C11—Fe1—C20	118.33 (14)	C21—Fe2—C23	69.24 (12)
C12—Fe1—C16	116.50 (14)	C22—Fe2—C29	104.93 (13)
C11—Fe1—C16	109.31 (13)	C21—Fe2—C29	126.15 (13)
C20—Fe1—C16	40.45 (16)	C23—Fe2—C29	116.60 (13)
C12—Fe1—C15	69.60 (12)	C22—Fe2—C30	122.93 (12)
C11—Fe1—C15	41.31 (12)	C21—Fe2—C30	162.30 (13)
C20—Fe1—C15	110.58 (15)	C23—Fe2—C30	104.56 (13)
C16—Fe1—C15	132.23 (14)	C29—Fe2—C30	40.51 (14)
C12—Fe1—C17	106.62 (13)	C22—Fe2—C28	118.92 (13)
C11—Fe1—C17	129.70 (14)	C21—Fe2—C28	109.34 (13)
C20—Fe1—C17	67.73 (16)	C23—Fe2—C28	152.21 (14)
C16—Fe1—C17	40.04 (15)	C29—Fe2—C28	40.39 (14)
C15—Fe1—C17	169.75 (14)	C30—Fe2—C28	67.99 (14)
C12—Fe1—C18	127.52 (14)	C22—Fe2—C26	160.83 (13)
C11—Fe1—C18	167.50 (14)	C21—Fe2—C26	156.59 (13)
C20—Fe1—C18	67.81 (16)	C23—Fe2—C26	124.57 (13)
C16—Fe1—C18	67.58 (15)	C29—Fe2—C26	67.88 (14)
C15—Fe1—C18	149.49 (14)	C30—Fe2—C26	40.26 (13)
C17—Fe1—C18	40.26 (14)	C28—Fe2—C26	67.91 (14)
C12—Fe1—C19	166.25 (15)	C22—Fe2—C25	69.49 (12)
C11—Fe1—C19	151.34 (14)	C21—Fe2—C25	41.26 (12)
C20—Fe1—C19	40.32 (16)	C23—Fe2—C25	68.34 (12)
C16—Fe1—C19	67.55 (16)	C29—Fe2—C25	165.84 (13)
C15—Fe1—C19	118.39 (14)	C30—Fe2—C25	153.43 (13)
C17—Fe1—C19	67.40 (15)	C28—Fe2—C25	129.93 (13)
C18—Fe1—C19	40.07 (15)	C26—Fe2—C25	121.49 (13)
C12—Fe1—C13	40.67 (12)	C22—Fe2—C27	155.37 (13)
C11—Fe1—C13	68.95 (12)	C21—Fe2—C27	122.29 (13)
C20—Fe1—C13	168.49 (16)	C23—Fe2—C27	163.39 (13)
C16—Fe1—C13	148.54 (15)	C29—Fe2—C27	67.98 (13)
C15—Fe1—C13	68.30 (12)	C30—Fe2—C27	67.90 (13)
C17—Fe1—C13	115.49 (14)	C28—Fe2—C27	40.50 (14)
C18—Fe1—C13	106.97 (14)	C26—Fe2—C27	40.34 (13)
C19—Fe1—C13	129.26 (15)	C25—Fe2—C27	111.37 (13)
C12—Fe1—C14	68.79 (13)	C22—Fe2—C24	68.99 (12)
C11—Fe1—C14	68.92 (12)	C21—Fe2—C24	68.85 (12)
C20—Fe1—C14	131.52 (16)	C23—Fe2—C24	40.44 (12)
C16—Fe1—C14	170.60 (15)	C29—Fe2—C24	151.27 (13)
C15—Fe1—C14	40.52 (12)	C30—Fe2—C24	117.84 (13)
C17—Fe1—C14	148.11 (14)	C28—Fe2—C24	166.87 (14)
C18—Fe1—C14	116.18 (14)	C26—Fe2—C24	108.25 (13)
C19—Fe1—C14	109.27 (15)	C25—Fe2—C24	40.31 (12)
C13—Fe1—C14	40.33 (13)	C27—Fe2—C24	128.60 (13)
C11—P1—C121	104.37 (13)	C21—P2—C221	105.06 (14)
C11—P1—C111	105.12 (14)	C21—P2—C211	106.42 (14)
C121—P1—C111	105.35 (13)	C221—P2—C211	103.73 (13)
C11—P1—S1	116.95 (10)	C21—P2—S2	115.91 (10)
C121—P1—S1	112.09 (11)	C221—P2—S2	111.95 (10)
C111—P1—S1	111.98 (11)	C211—P2—S2	112.76 (11)
C1—O1—C3	112.1 (2)	C4—O2—C2	111.1 (2)
C15—C11—C12	107.3 (3)	C25—C21—C22	107.3 (3)
C15—C11—P1	124.7 (2)	C25—C21—P2	125.1 (2)
C12—C11—P1	127.9 (2)	C22—C21—P2	127.3 (2)
C15—C11—Fe1	70.01 (17)	C25—C21—Fe2	70.00 (16)
C12—C11—Fe1	68.98 (16)	C22—C21—Fe2	68.83 (16)
P1—C11—Fe1	129.02 (16)	P2—C21—Fe2	130.76 (16)
C13—C12—C11	107.5 (3)	C23—C22—C21	107.3 (3)
C13—C12—C1	126.0 (3)	C23—C22—C2	124.5 (3)
C11—C12—C1	126.5 (3)	C21—C22—C2	128.2 (3)
C13—C12—Fe1	70.73 (17)	C23—C22—Fe2	70.04 (17)
C11—C12—Fe1	69.16 (16)	C21—C22—Fe2	69.46 (16)
C1—C12—Fe1	125.5 (2)	C2—C22—Fe2	125.2 (2)
C12—C13—C14	109.0 (3)	C24—C23—C22	108.6 (3)
C12—C13—Fe1	68.60 (17)	C24—C23—Fe2	70.32 (18)
C14—C13—Fe1	70.25 (19)	C22—C23—Fe2	68.80 (16)
C12—C13—H13	125.5	C24—C23—H23	125.7
C14—C13—H13	125.5	C22—C23—H23	125.7
Fe1—C13—H13	127.3	Fe2—C23—H23	126.8
C13—C14—C15	108.1 (3)	C25—C24—C23	108.6 (3)
C13—C14—Fe1	69.42 (18)	C25—C24—Fe2	69.65 (17)
C15—C14—Fe1	69.03 (18)	C23—C24—Fe2	69.24 (18)
C13—C14—H14	126.0	C25—C24—H24	125.7
C15—C14—H14	126.0	C23—C24—H24	125.7
Fe1—C14—H14	127.2	Fe2—C24—H24	127.0
C14—C15—C11	108.1 (3)	C24—C25—C21	108.2 (3)
C14—C15—Fe1	70.45 (17)	C24—C25—Fe2	70.04 (17)
C11—C15—Fe1	68.68 (16)	C21—C25—Fe2	68.74 (16)
C14—C15—H15	125.9	C24—C25—H25	125.9
C11—C15—H15	125.9	C21—C25—H25	125.9
Fe1—C15—H15	126.5	Fe2—C25—H25	126.9
C17—C16—C20	108.3 (4)	C30—C26—C27	108.3 (3)
C17—C16—Fe1	70.3 (2)	C30—C26—Fe2	69.80 (18)
C20—C16—Fe1	69.7 (2)	C27—C26—Fe2	70.00 (18)
C17—C16—H16	125.9	C30—C26—H26	125.8
C20—C16—H16	125.9	C27—C26—H26	125.8
Fe1—C16—H16	125.8	Fe2—C26—H26	126.0
C16—C17—C18	108.1 (3)	C26—C27—C28	107.7 (3)
C16—C17—Fe1	69.7 (2)	C26—C27—Fe2	69.66 (18)
C18—C17—Fe1	69.93 (19)	C28—C27—Fe2	69.54 (19)
C16—C17—H17	125.9	C26—C27—H27	126.2
C18—C17—H17	125.9	C28—C27—H27	126.2
Fe1—C17—H17	126.0	Fe2—C27—H27	126.2
C19—C18—C17	107.8 (3)	C29—C28—C27	108.0 (3)
C19—C18—Fe1	70.0 (2)	C29—C28—Fe2	69.74 (19)
C17—C18—Fe1	69.81 (19)	C27—C28—Fe2	69.97 (19)
C19—C18—H18	126.1	C29—C28—H28	126.0
C17—C18—H18	126.1	C27—C28—H28	126.0
Fe1—C18—H18	125.7	Fe2—C28—H28	125.9
C18—C19—C20	108.3 (3)	C28—C29—C30	108.0 (3)
C18—C19—Fe1	69.9 (2)	C28—C29—Fe2	69.86 (19)
C20—C19—Fe1	69.4 (2)	C30—C29—Fe2	69.78 (18)
C18—C19—H19	125.9	C28—C29—H29	126.0
C20—C19—H19	125.9	C30—C29—H29	126.0
Fe1—C19—H19	126.4	Fe2—C29—H29	125.9
C19—C20—C16	107.6 (3)	C26—C30—C29	108.0 (3)
C19—C20—Fe1	70.3 (2)	C26—C30—Fe2	69.94 (18)
C16—C20—Fe1	69.9 (2)	C29—C30—Fe2	69.71 (18)
C19—C20—H20	126.2	C26—C30—H30	126.0
C16—C20—H20	126.2	C29—C30—H30	126.0
Fe1—C20—H20	125.2	Fe2—C30—H30	125.9
O1—C1—C12	108.2 (2)	O2—C2—C22	109.5 (2)
O1—C1—H36A	110.0	O2—C2—H41A	109.8
C12—C1—H36A	110.0	C22—C2—H41A	109.8
O1—C1—H36B	110.0	O2—C2—H41B	109.8
C12—C1—H36B	110.0	C22—C2—H41B	109.8
H36A—C1—H36B	108.4	H41A—C2—H41B	108.2
O1—C3—C311	107.6 (2)	O2—C4—C411	109.6 (2)
O1—C3—H37A	110.2	O2—C4—H42A	109.8
C311—C3—H37A	110.2	C411—C4—H42A	109.8
O1—C3—H37B	110.2	O2—C4—H42B	109.8
C311—C3—H37B	110.2	C411—C4—H42B	109.8
H37A—C3—H37B	108.5	H42A—C4—H42B	108.2
C112—C111—C116	119.0 (3)	C212—C211—C216	119.3 (3)
C112—C111—P1	122.6 (2)	C212—C211—P2	120.1 (2)
C116—C111—P1	118.3 (2)	C216—C211—P2	120.4 (2)
C111—C112—C113	120.0 (3)	C211—C212—C213	119.7 (3)
C111—C112—H112	120.0	C211—C212—H212	120.2
C113—C112—H112	120.0	C213—C212—H212	120.2
C114—C113—C112	120.4 (3)	C214—C213—C212	120.5 (3)
C114—C113—H113	119.8	C214—C213—H213	119.7
C112—C113—H113	119.8	C212—C213—H213	119.7
C115—C114—C113	119.9 (3)	C215—C214—C213	120.0 (3)
C115—C114—H114	120.1	C215—C214—H214	120.0
C113—C114—H114	120.1	C213—C214—H214	120.0
C114—C115—C116	120.1 (3)	C214—C215—C216	120.2 (3)
C114—C115—H115	119.9	C214—C215—H215	119.9
C116—C115—H115	119.9	C216—C215—H215	119.9
C115—C116—C111	120.5 (3)	C215—C216—C211	120.3 (3)
C115—C116—H116	119.7	C215—C216—H216	119.8
C111—C116—H116	119.7	C211—C216—H216	119.8
C126—C121—C122	119.3 (3)	C226—C221—C222	118.8 (3)
C126—C121—P1	121.7 (2)	C226—C221—P2	122.3 (2)
C122—C121—P1	119.0 (2)	C222—C221—P2	118.9 (2)
C123—C122—C121	120.3 (3)	C223—C222—C221	121.0 (3)
C123—C122—H122	119.8	C223—C222—H222	119.5
C121—C122—H122	119.8	C221—C222—H222	119.5
C122—C123—C124	120.1 (3)	C222—C223—C224	119.5 (3)
C122—C123—H123	120.0	C222—C223—H223	120.2
C124—C123—H123	120.0	C224—C223—H223	120.2
C125—C124—C123	120.0 (3)	C225—C224—C223	120.4 (3)
C125—C124—H124	120.0	C225—C224—H224	119.8
C123—C124—H124	120.0	C223—C224—H224	119.8
C124—C125—C126	120.1 (3)	C224—C225—C226	120.2 (3)
C124—C125—H125	120.0	C224—C225—H225	119.9
C126—C125—H125	120.0	C226—C225—H225	119.9
C125—C126—C121	120.1 (3)	C225—C226—C221	120.1 (3)
C125—C126—H126	119.9	C225—C226—H226	119.9
C121—C126—H126	119.9	C221—C226—H226	119.9
C312—C311—C316	118.0 (3)	C416—C411—C412	118.0 (3)
C312—C311—C3	120.2 (3)	C416—C411—C4	120.7 (3)
C316—C311—C3	121.7 (3)	C412—C411—C4	121.3 (3)
C311—C312—C313	121.3 (3)	C413—C412—C411	120.6 (3)
C311—C312—H32	119.3	C413—C412—H44	119.7
C313—C312—H32	119.3	C411—C412—H44	119.7
C312—C313—C314	120.8 (3)	C412—C413—C414	121.6 (3)
C312—C313—H33	119.6	C412—C413—H45	119.2
C314—C313—H33	119.6	C414—C413—H45	119.2
C313—C314—C315	117.8 (3)	C415—C414—C413	117.2 (3)
C313—C314—C317	120.7 (3)	C415—C414—C417	122.0 (3)
C315—C314—C317	121.5 (3)	C413—C414—C417	120.8 (3)
C316—C315—C314	121.1 (3)	C416—C415—C414	121.9 (3)
C316—C315—H35	119.4	C416—C415—H200	119.0
C314—C315—H35	119.4	C414—C415—H200	119.0
C315—C316—C311	120.9 (3)	C415—C416—C411	120.6 (3)
C315—C316—H38	119.5	C415—C416—H100	119.7
C311—C316—H38	119.5	C411—C416—H100	119.7
C314—C317—H30A	109.5	C414—C417—H40A	109.5
C314—C317—H30B	109.5	C414—C417—H40B	109.5
H30A—C317—H30B	109.5	H40A—C417—H40B	109.5
C314—C317—H30C	109.5	C414—C417—H40C	109.5
H30A—C317—H30C	109.5	H40A—C417—H40C	109.5
H30B—C317—H30C	109.5	H40B—C417—H40C	109.5

### Hydrogen-bond geometry (Å, °)

**Table:**

Cg1 and Cg2 are the centroids of the C111–C116 and C211–C216 rings, respectively.

**Table:**

*D*—H···*A*	*D*—H	H···*A*	*D*···*A*	*D*—H···*A*
C312—H32···Cg1	0.95	2.82	3.703 (3)	156
C123—H123···Cg2^i^	0.95	2.91	3.741 (4)	147

Symmetry codes: (i) *x*, *y*+1, *z*.
